# Immunoglobulin G4 hypophysitis in a 63-year-old woman with no autoimmune history: a case report

**DOI:** 10.1186/s13256-021-03018-7

**Published:** 2021-09-05

**Authors:** Zachary C. Gersey, Kenan R. Rajjoub, Thomas M. Pearce, Scott A. Segel, Paul A. Gardner, Carl H. Snyderman, Eric W. Wang, Georgios A. Zenonos

**Affiliations:** 1 Department of Neurological Surgery, University of Pittsburgh Medical Center, 200 Lothrop St Suite B-400, Pittsburgh, PA 15213 USA; 2 Department of Neurological Surgery, The George Washington University School of Medicine, Washington, District of Columbia, USA; 3 Division of Anatomic Pathology, Department of Pathology, University of Pittsburgh Medical Center, Pittsburgh, PA USA; 4 Department of Endocrinology, University of Pittsburgh Medical Center, Pittsburgh, PA USA; 5 Department of Otolaryngology, University of Pittsburgh School of Medicine, Pittsburgh, PA USA

**Keywords:** IgG4, Hypophysitis, Endonasal, Skull base, Case report

## Abstract

**Background:**

Immunoglobulin-G4-related hypophysitis is a rare inflammatory disease that can present as a tumefactive pituitary lesion mimicking hypophyseal neoplasms such as pituitary adenoma or craniopharyngioma. The literature on this entity is sparse, with fewer than 100 cases reported across 19 publications; a recent review found only 24 cases published from 2007 to 2018. Previous reports have described demographic differences, with immunoglobulin-G4-related hypophysitis in females tending to present in the second and third decades in association with other autoimmune disease, while males tend to present in the fifth and sixth decades of life without an autoimmune history.

**Case presentation:**

In contrast to the reported demographic trends, here we describe a unique case of immunoglobulin-G4-related hypophysitis in a 63-year-old white female with no history of autoimmune disease who presented with a rapidly enlarging sellar and hypothalamic mass causing headaches and cranial nerve palsies, prompting biopsy for diagnosis. The patient experienced rapid response to treatment with high-dose steroids and rituximab.

**Conclusion:**

The case contributes to the growing clinicopathologic description of immunoglobulin-G4-related hypophysitis and illustrates that this diagnosis should be a consideration even outside the conventional demographic setting.

## Introduction

Primary hypophysitis is a rare disease, accounting for ~ 0.4% of all diagnosed pituitary lesions, and it may masquerade clinically as a mass lesion suggestive of a neoplastic process on radiologic studies [29]. A definitive tissue diagnosis of primary hypophysitis is made on biopsy by documentation of an inflammatory infiltrate in the absence of a secondary background process such as a neoplasm, infection, or systemic inflammatory condition. Histologically, primary hypophysitis can be categorized by the cellular composition of the inflammatory infiltrate as granulomatous, xanthomatous, lymphocytic, or plasmacytic. When a predominantly plasmacytic infiltrate contains a high proportion of immunoglobulin G4 (IgG4)-positive cells, a diagnosis of IgG4-related hypophysitis (IgG4-RH) can be made [29]. However, clinicopathologic criteria for diagnosis of IgG4-RH are not well established, particularly in the absence of systemic involvement or elevated plasma IgG4 levels [2, 7].

IgG4-related disease (IgG4-RD) is a fibroinflammatory process that can involve multiple organs, less commonly the pituitary, and is often but not always associated with elevated serum IgG4 levels [7]. If there is involvement of the pituitary gland, the tumefactive lesions caused by IgG4-RD mimic hypophyseal tumors and may be mistaken for an adenoma or craniopharyngioma. Previous case series have described gender differences in the presentation of IgG4-RH, with males tending to present later in life with elevated serum IgG4 levels and systemic involvement, and females presenting in the second or third decade of life with isolated lesions and normal serum IgG4 levels, and often in conjunction with autoimmune diseases [29]. However, the literature on this condition remains very limited, and the spectrum of clinical presentation is not well defined.

In this case report, we present a unique case of IgG4 hypophysitis that will add to the medical literature. The presented case is an important addition to the literature as it is not a classic presentation of IgG4 hypophysitis since the patient had no autoimmune history, normal IgG4 serum levels, and is outside the typical age range for this disease. This case report will serve as an aid for physicians and surgeons to improve the timely diagnosis and treatment of this rare entity.

## Case description

**Fig. 1:**
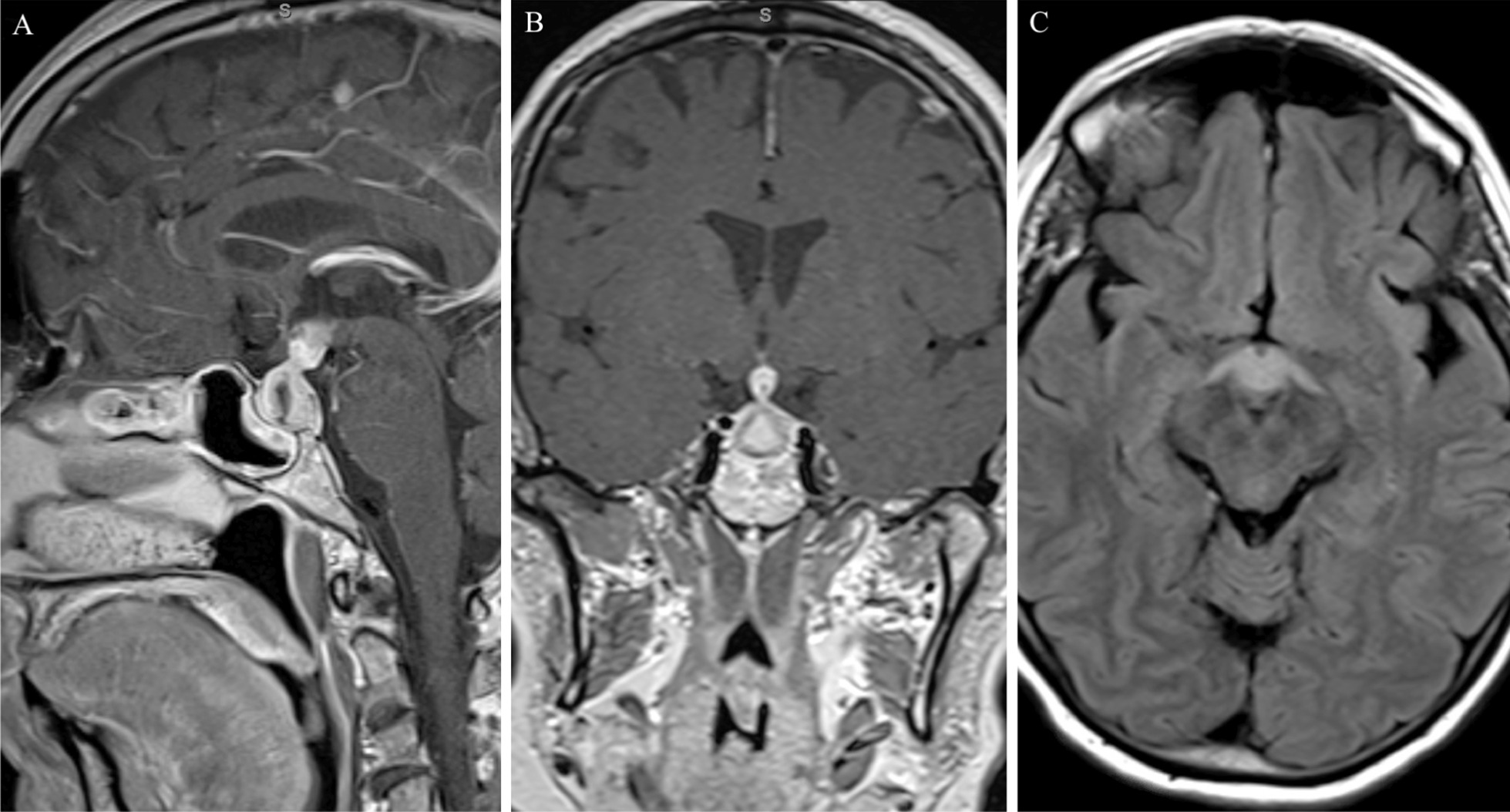
MRI brain with and without gadolinium on initial presentation. Sagittal T1 post-gadolinium (**A**). Coronal T1 post-gadolinium (**B**). Axial T2 FLAIR (**C**)

**Fig. 2:**
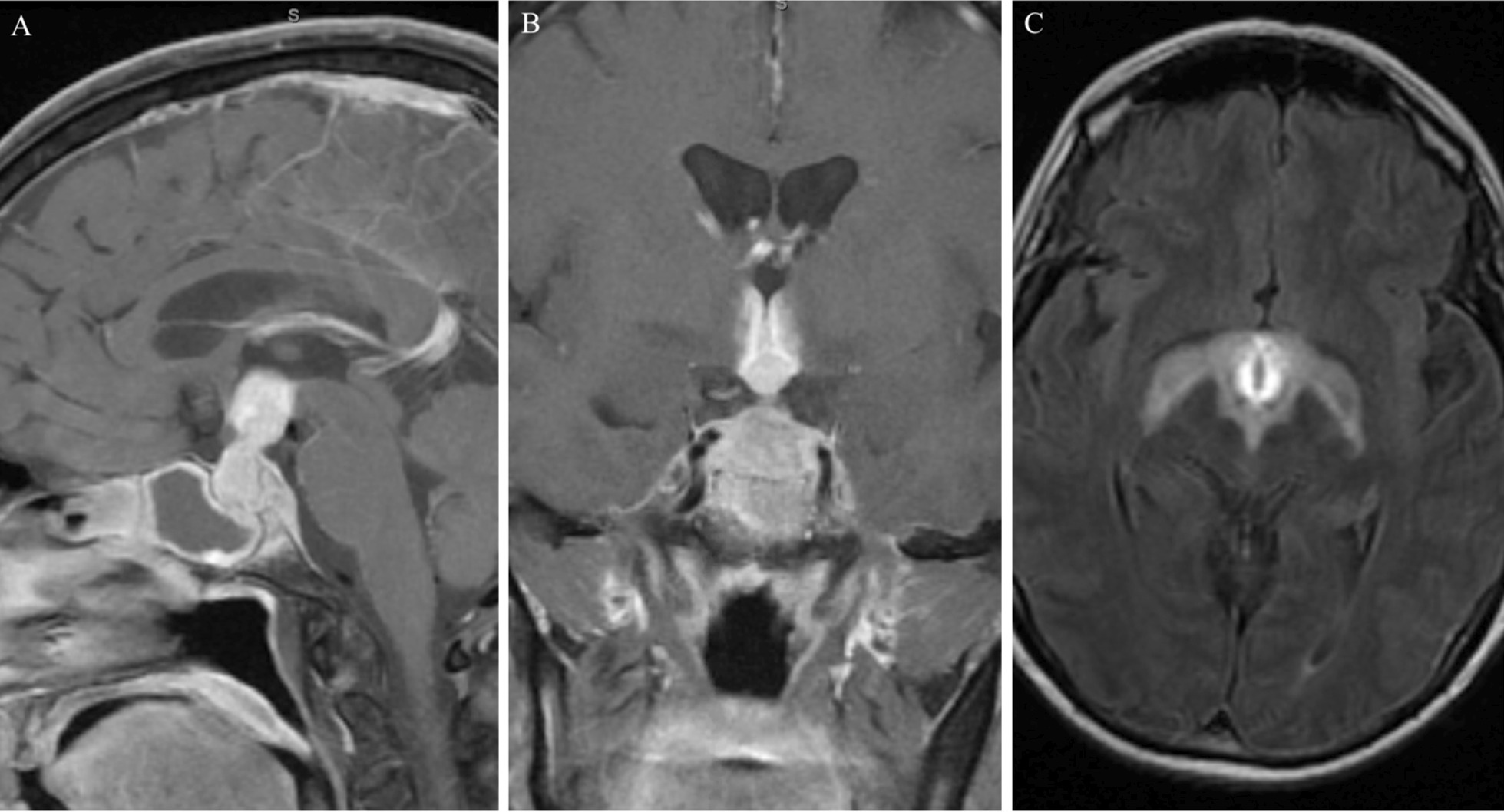
MRI brain with and without gadolinium 1 month after initial presentation. Sagittal T1 post-gadolinium (**A**). Coronal T1 post-gadolinium (**B**). Axial T2 FLAIR** C**

A 63-year-old white female with a past medical history of hypertension and previous appendectomy presented after 2 months of progressive headaches, with magnetic resonance imaging (MRI) demonstrating a sellar and suprasellar mass that grew rapidly in 1 month’s time (Figs. 1 and 2). The patient originally reported holocephalic headaches, with blurry vision and diplopia (more apparent with left lateral gaze). She described the headaches as moderate to severe in intensity, and characterized the pain as varying between sharp and throbbing. In addition, she reported significant fatigue as well as polydipsia and polyuria (awakening five to six times per night to urinate and drink water). The patient was a retired banker and lived at home with her husband and therefore had no social or environmental exposures and had no family history of autoimmune or neurological diseases. She denied any changes in ring/shoe size, muscle weakness, weight gain/loss, abnormal hair growth, chest pain, shortness of breath, difficulty swallowing, language changes, focal weakness, numbness, tingling, or other neurologic or systemic symptoms. At the time of presentation, the patient was taking hydrochlorothiazide–triamterene and ramipril but was not on any immune-modulating medications. She was a former smoker and consumed three to four glasses of wine per week.

On presentation, the patient was afebrile with a normal pulse (69 beats per minute) and normal blood pressure (124/70 mmHg). On physical examination, she was alert, cooperative, and oriented to person, location, and date. She had a visual acuity of 20/25 in the right eye and 20/20 in the left with a partial left-sided cranial nerve VI palsy. She was otherwise neurologically intact. Her neck was supple and nontender, oral mucosa moist, lungs clear to auscultation, heart rate normal and with a regular rhythm, and her skin warm and dry.

Initial outpatient laboratory studies showed an elevated antinuclear antibody (ANA) of 1:160 (normal: negative) and normal inflammatory testing [erythrocyte sedimentation rate (ESR)/C-reactive protein (CRP)]. Laboratory studies showed an 7 am serum cortisol 28.1 μg/dL (slightly elevated), concurrent plasma adrenocorticotropic hormone (ACTH) 29 pg/mL (normal), free triiodothyronine (T3) 2.0 pg/m (normal 2.3–4.2 pg/m), follicle-stimulating hormone (FSH) 12.3 mIU/mL, luteinizing hormone (LH) 0.4 IU/L (low), Insulin-like growth factor 1 (IGF-I) 154 ng/mL, prolactin of 33.3 ng/mL (normal less than 20.3 ng/mL for a menopausal woman), and IgG4 levels of 57.1 mg/dL (7–89 mg/dL normal). Lumbar puncture was obtained, which showed no organisms, white blood cell count (WBC) of 95 cells/cu mm, red blood cell count (RBC) of 4 cells/cu mm, 54% neutrophils, 42% lymphocytes, 55 mg/dL glucose, 154 mg/dL protein, and IgG 26.7 mg/dL. Repeat laboratory studies 1 month later showed a 7 am cortisol level of 2 ug/dL and free thyroxine (T4) level of 2.9 ug/dL (normal range is 5–12). ACTH, growth hormone (GH), LH, FSH, thyroid-stimulating hormone (TSH), and prolactin levels were within normal limits. Her serum sodium was within normal limits (value), pointing to compensated diabetes insipidus. A water deprivation test was deferred at this time.

An MRI of the brain with and without gadolinium was obtained at initial presentation and showed a 1.5 × 1.2 × 1.4 cm enhancing lesion involving the sella and adjacent structures, including the infundibulum, hypothalamus, and sphenoid sinus (Fig. 1A, B). T2 FLAIR also showed edema extending symmetrically along the bilateral hypothalami and fornices (Fig. 1C). At that time, the patient was managed conservatively with observation. Repeat MRI 1 month later after her symptoms continued to progress showed progression of the lesion, now measuring 3.2 × 2.2 × 2.0 cm with more extension into the bilateral hypothalami and fornices and sphenoid sinus as well as new extension into the clivus with anterior displacement of the optic chiasm (Fig. 2A, B). T2 FLAIR demonstrated more extensive edema into the hypothalami and fornices (Fig. 2C). Notably, there was also diffusion restricting content filling the sphenoid sinus with peripheral enhancement concerning for infection. CT of the chest, abdomen, and pelvis with contrast showed no evidence of malignancy. Due to the progression of disease, the patient was transferred to our institution for further workup.

**Fig. 3:**
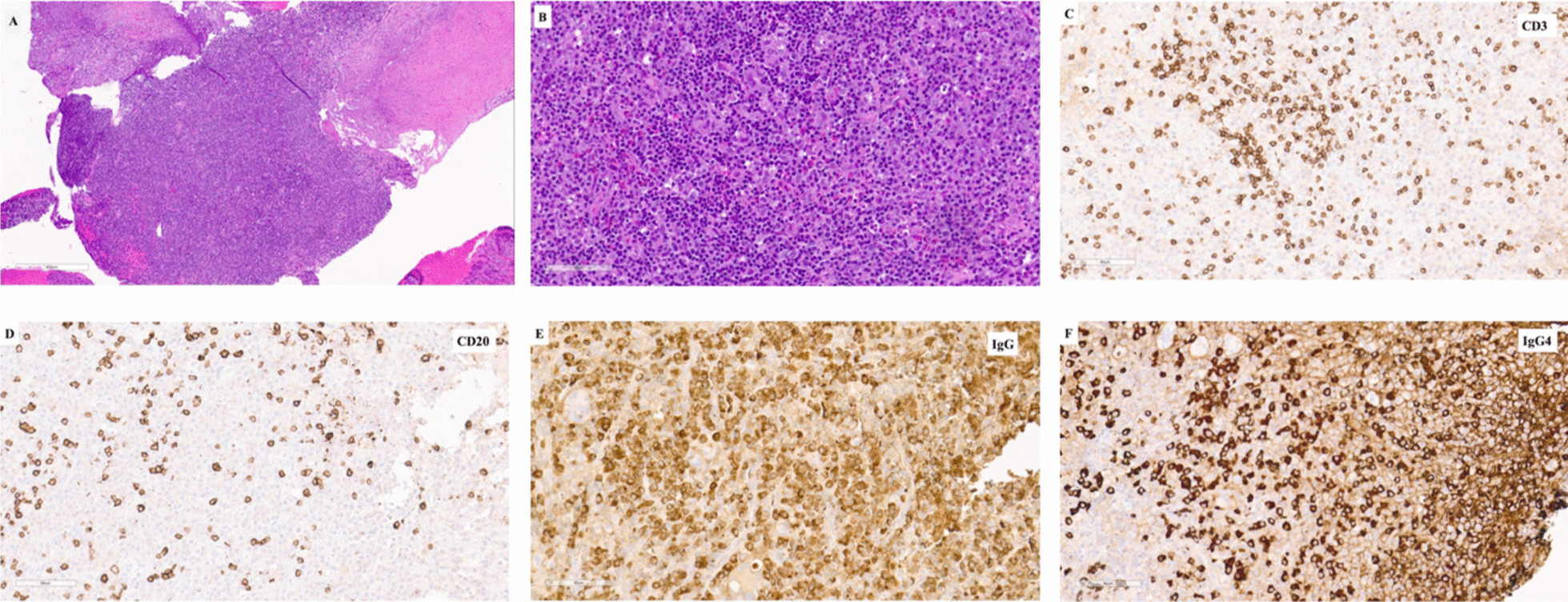
Histopathologic findings. Hematoxylin and eosin (H&E)-stained sections demonstrate a densely cellular infiltrate with regions of fibrosis (**A**). The cellular areas are composed of lymphocytes, plasma cells, histiocytes, and eosinophils (**B**). Immunohistochemical stains demonstrate scattered CD3-positive T cells (**C**) and CD20-positive B cells (**D**), and abundant IgG-positive plasma cells (**E**), many of which are positive for IgG4 (**F**)

An endoscopic endonasal biopsy was performed 6 weeks after initial presentation. Endoscopically, it was noted that the fluid filling the sphenoid sinus was inspissated. A biopsy of the sphenoid sinus mucosa was evaluated intraoperatively by frozen section, which demonstrated acute and chronic inflammation, necrosis, and possible poorly formed granulomas. This finding raised the possibility of an autoimmune process such as granulomatous vasculitis or a chronic infection. A biopsy of the sellar lesion, which was performed through an infrasellar approach, was also evaluated intraoperatively; cytologic preparations showed predominantly chronic inflammation with occasional multinucleated giant cells. Permanent sections demonstrated an intense chronic inflammatory infiltrate composed predominantly of plasma cells, histiocytes, and lymphocytes (Fig. 3A–B). Numerous eosinophils were present, but there were few, if any, neutrophils. Immunohistochemical stains demonstrated abundant IgG-positive plasma cells, more than 40% of which stained positively for IgG4, and a smaller mixed T- and B-cell lymphoid population (Fig. 3C–F). The endoscopically sampled tissue fragments did not show histologic evidence of classic storiform fibrosis or obliterative phlebitis. Additional studies were performed to exclude infectious and neoplastic processes. Together, the findings were consistent with a diagnosis of IgG4-related hypophysitis. Notably, the cultures from the inspissated contents of the sphenoid sinus grew normal sinus flora but predominantly *Cutibacterium acnes*, a microbe that is not normally part of local flora. Notably, the sellar floor was partially dehiscent, and the sellar inflammatory tissue was biopsied very focally just in that area to avoid spread of any infection intracranially. Final pathology did not reveal any organisms, so it is thought that the sinusitis was not due to an infection, but the patient did complete Augmentin 875 mg twice daily for 7 days while we awaited the pathology report.

**Fig. 4:**
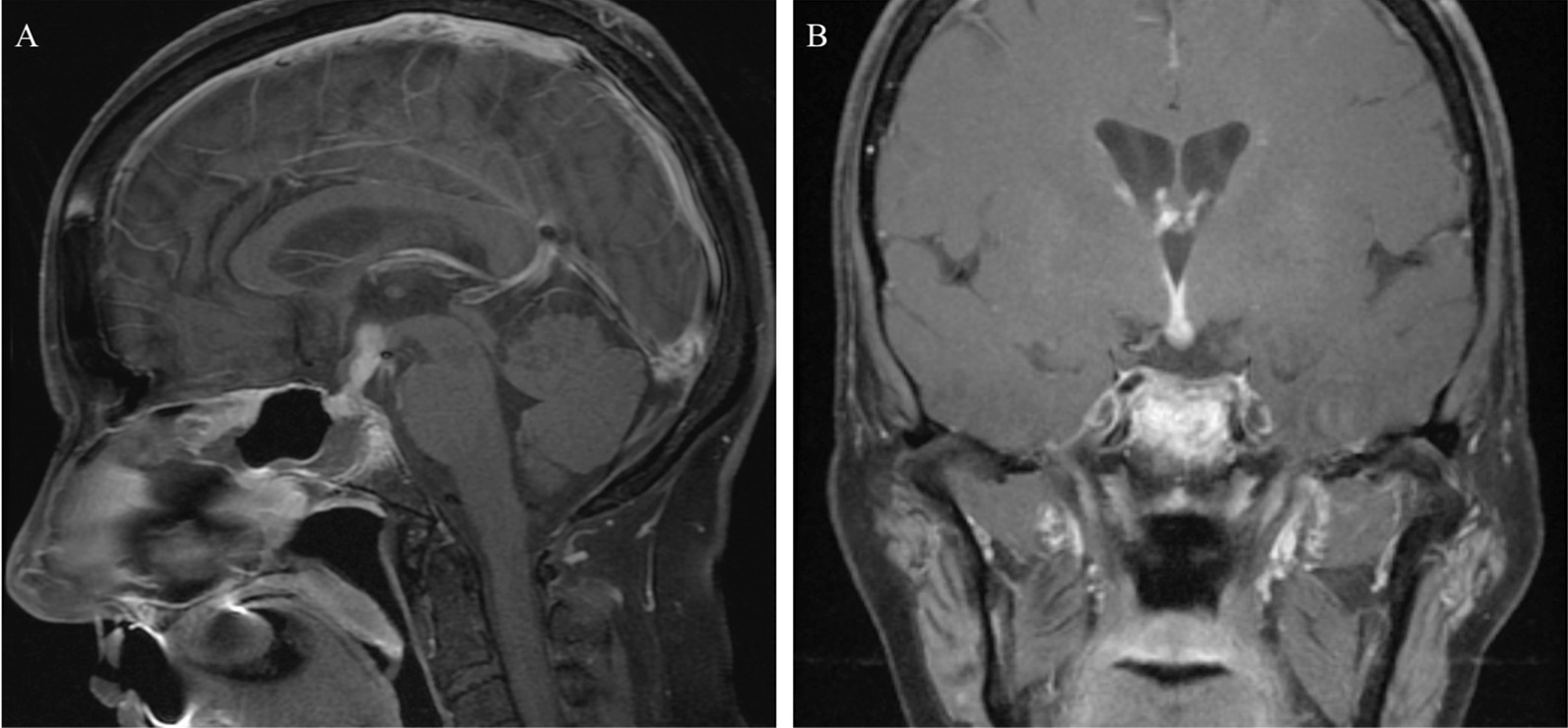
MRI brain with and without gadolinium 5 days post-biopsy and high-dose steroids. Sagittal T1 post-gadolinium (**A**). Coronal T1 post-gadolinium (**B**)

**Fig. 5:**
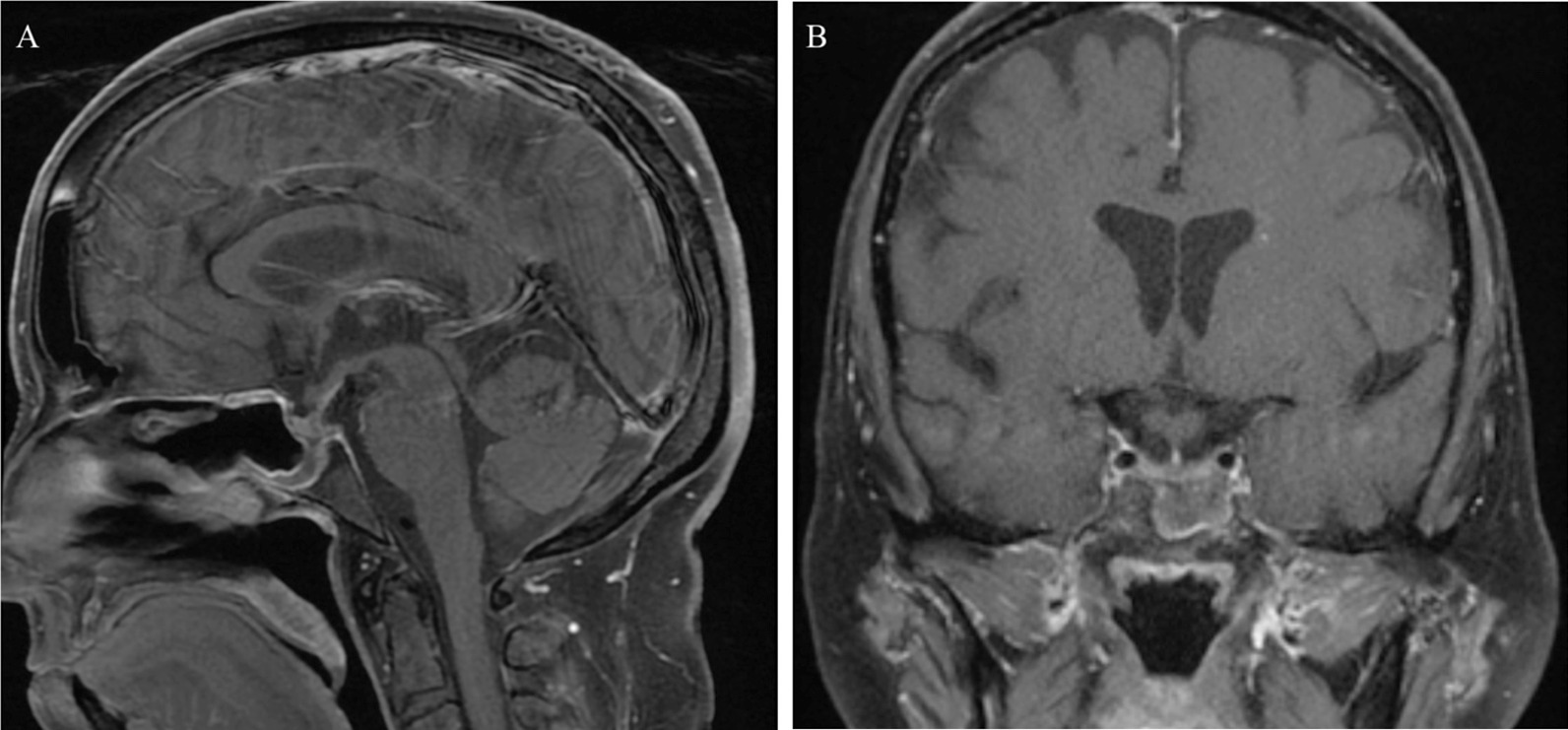
MRI brain with and without gadolinium 3 months post-biopsy showing complete resolution of abnormal enhancement and return to normal size of pituitary gland and stalk. Sagittal T1 post-gadolinium (**A**). Coronal T1 post-gadolinium (**B**)

The patient was treated with levothyroxine (88 μg oral tablet daily) for central hypothyroidism, high-dose prednisone (tapered over 1 year), and rituximab (1 g intravenously every 6 months, planned for 2 years) postoperatively. Repeat MRI 5 days after biopsy showed significant improvement in the sellar and suprasellar enhancing lesion (Fig. 4A, B). At the time of discharge 6 days later, her cranial nerve (CN) VI palsy persisted, but her headache and blurry vision had resolved. The patient was followed for 3 months while continuing her prednisone and rituximab, and on her outpatient clinic appointment, the CN VI palsy had resolved. At that time, the imaging showed complete resolution of the abnormal enhancement in and around the hypophysis with a return to normal size of the pituitary gland, and stalk had returned to normal size (Fig. 5A, B). She continues to have central hypothyroidism and diabetes insipidus for which she remains on levothyroxine (88 μg oral tablet daily) and desmopressin (0.05 mg in the morning and 0.1 mg in the evening by mouth). At 1 year follow-up, the patient remained asymptomatic and had no evidence of pituitary inflammation on her MRI at that time.

## Discussion

Here we describe a unique case of IgG4 hypophysitis. The patient was outside the “normal age-range,” had no autoimmune history, and had normal serum levels of IgG4. All of these attributes make this an interesting case that will contribute to the literature and assist physicians and surgeons in the future in the diagnosis and management of this rare but potentially debilitating disease.

Goudie and Pinkerton in 1962 were the first to report autoimmune hypophysitis in a 22-year-old female who developed amenorrhea and postpartum hypothyroidism [10]. On autopsy, it was later found that she had lymphocytic thyroiditis, atrophic adrenals, and an atrophied lymphocytic pituitary gland, suggesting systemic autoimmune disease. Subsequent reports made similar observations in relation to hypophyseal inflammatory diseases [1, 8, 9, 15, 18, 20] with pituitary size ranging from atrophic to enlarged. Any part of the pituitary is susceptible; there is even potential for extension into the cavernous sinus [25]. IgG4-RH is a recently recognized immune-mediated hypophysitis that is distinct from lymphocytic, xanthomatous, and granulomatous hypophysitis, and thought to be related to IgG4-RD seen in other organs [21]. Recognition of this disease is important, because timely treatment of IgG4-RD during the initial inflammatory phase can prevent the permanent organ damage caused by progressive destructive fibrosis.

Presentation of hypophysitis can vary; however, it typically has a component of hypopituitarism and sometimes diabetes insipidus, combined with mass effect from the increased size [8]. Common presenting symptoms are headaches, fatigue, nausea, vomiting, weight loss, endocrine dysfunction, and visual disturbances [19, 2, 3, 29, 30, 32]. Both lymphocytic hypophysitis and IgG4-RH may be associated with other autoimmune diseases, particularly in female patients. Previously documented autoimmune diseases associated with lymphocytic hypophysitis are Hashimoto’s thyroiditis [5, 10, 26], Grave’s diseases [1, 6, 29], Sjogren’s syndrome [22], Addison’s disease [28], primary biliary cirrhosis [24], diabetes mellitus type 1 [14], systemic lupus erythematosus [12, 13], atrophic gastritis [10, 23], pernicious anemia [15], autoimmune polyendocrine syndrome type 1/autoimmune polyendocrinopathy–candidiasis–ectodermal dystrophy (APECED) [31], and autoimmune hepatitis [27].

The demographic characteristics of patients with IgG4-RH are not entirely clear, due to the paucity of reports of this entity. Of the 24 cases documented between 2007 and 2018, 12 patients were male and 12 female. Males and females in this cohort demonstrated different age of onset for this disease: females presented at a mean age of 28.5 years (range 14–55 years), compared with males, who presented at a mean age of 62.5 years (range 40–77 years) [29].

Our patient is a 63-year-old white female with IgG4-RH that presented as a rapidly enlarging pituitary and infundibular mass. Since she has no past medical history of autoimmune disease and is not in the typical age range for female patients, her case is unusual and represents a unique clinical scenario that has, to our knowledge, not yet been reported [29]. Our patient’s later-in-life onset and the fact that she had no autoimmune medical history supports the growing body of evidence that IgG4-RH, even without systemic involvement or elevated serum IgG4 levels, is a distinct entity related to IgG4-RD more broadly, and not a subset of lymphocytic hypophysitis. In addition, in older patients with an atypical demographic such as this one, the suspicion of lymphoma or metastasis is raised, significantly lowering the threshold for biopsy.

The concomitant sphenoid sinus infection, predominantly caused by *C. acnes*, is an odd phenomenon; it is unclear whether a protracted low-grade infection by this microbe could have triggered the hypophysitis. It is conceivable though that, as with many autoimmune diseases, an infection could be the initiating factor. Alternatively, the sinusitis may have been the result of the florid adjacent inflammation causing an obstruction in outflow and ensuing infection. Obviously, more case studies are needed to further investigate this hypothesis.

Pituitary involvement by IgG4-RD is a diagnostic challenge, especially in the absence of other organ involvement or elevated serum IgG4 levels. Criteria for diagnosing IgG4-RH on pituitary biopsy are currently not well defined. Suggestive histologic features include an elevated ratio of IgG4+/IgG+ plasma cells coupled with a minimum number of IgG4+ cells, but thresholds for the values of these metrics may be tissue-specific, and it is not clear that criteria created for other organ systems can be directly applied to the pituitary. It remains an open question whether the characteristic features of IgG4-RD seen in other locations, including storiform fibrosis and obliterative phlebitis, should be required for a definitive diagnosis. It is plausible that such features could develop over time, but the small physical confines of the sella combined with the functional importance of the involved structures may lead to earlier presentation of hypophysitis in comparison with other sites of involvement such as the retroperitoneum. Similarly, the limited bulk of disease in the hypophysis may be insufficient to generate elevated serum IgG4 levels in the absence of involvement of other organ systems. Although a recent literature review suggests that the IgG4+/IgG+ ratio alone may not be specific for IgG4-RH, and proposes the inclusion of other organ involvement and/or elevated serum IgG4 as diagnostic criteria, the issue is far from resolved [29].

IgG4-related hypophysitis, like lymphocytic hypophysitis, has historically been treated with corticosteroids owing to the similarity in presentation and histology to other autoimmune conditions. Postoperative treatment of IgG4-related sellar and suprasellar lesions has been varied. Most recently, there have been adjunctive postoperative biologic treatments that have been attempted. Corticosteroids were given in all cases; additionally, two patients received rituximab [3], one patient received azathioprine [4], and one patient received mycophenolate mofetil [11]. No death occurred in the cases presented [29]; however, there has been no comment in the literature about any residual deficits after treatment. In cases of IgG4 disease refractory to corticosteroids, rituximab alone has been used with promising results [16, 17]. Khosroshahi *et al*. suggest that rituximab’s depletion of CD20-positive B cells, the cells that differentiate into plasma cells that produce IgG4, is what makes it an effective therapy for IgG4-related disease [16].

In 2011, Leporati *et al.* proposed that surgical intervention for biopsy is not mandatory for diagnosis, assuming that serologic and radiographic evidence coupled with clinical suspicion is present. Radiographic evidence is limited by a lack of standardized radiographic features [21]. In more “textbook” cases of IgG4-RH, such as a patient matching expected demographic characteristics and with elevated serum IgG4 levels and/or systemic IgG4-RD, we do not recommend biopsy upfront, but instead suggest empiric treatment. If empiric treatment fails, or for atypical presentations, biopsy is recommended for a definitive tissue diagnosis.

## Conclusion

IgG4-RH remains a poorly understood clinical entity. This unusual case of IgG4-RH in a woman in her seventh decade of life with no past history of autoimmune disease, no systemic IgG4-RD, and normal serum IgG4 levels. Radiographically, she had a rapidly growing heterogeneous sellar lesion over a 1-month interval with signs and symptoms of mass effect. After biopsy of the sellar lesion demonstrated abundant IgG4+ plasma cells, she was treated with high-dose steroids and rituximab, leading to rapid symptomatic improvement and near-complete resolution of her imaging findings over the subsequent months. The case contributes to the growing clinicopathologic description of IgG4-RH and illustrates that this diagnosis should be a consideration even outside the conventional demographic setting.

## Limitations

Although our patient showed rapid improvement that was sustained over months, the long-term efficacy of the therapeutic regimen over several years remains unclear. Furthermore, despite the absence of any concomitant autoimmune disease in our patient, the possibility that hypophysitis was the initial presentation of a systemic condition that could manifest in a delayed fashion cannot be excluded.

## Data Availability

Data sharing not applicable to this article as no datasets were generated or analyzed during the current study.

## References

[1] Bayram F, Keleştimur F, Öztürk F, Selçuklu A, Patiroğlu TE, Beyhan Z. Lymphocytic hypophysitis in a patient with Graves’ disease. J Endocrinol Invest. 1998;21(3):193–7. 10.1007/BF03347301 9591217

[2] Bernreuther C, Illies C, Flitsch J, Buchfelder M, Buslei R, Glatzel M, Saeger W. IgG4-related hypophysitis is highly prevalent among cases of histologically confirmed hypophysitis. Brain Pathol. 2017;27(6):839–45. 10.1111/bpa.12459 PMC8029148 27864854

[3] Bullock DR, Miller BS, Clark HB, Hobday PM. Rituximab treatment for isolated IgG4-related hypophysitis in a teenage female. Endocrinol Diabetes Metab Case Rep. 2018. 10.1530/EDM-18-0135. PMC6311468 30601761

[4] Caputo C, Bazargan A, McKelvie PA, Sutherland T, Su CS, Inder WJ. Hypophysitis due to IgG4-related disease responding to treatment with azathioprine: an alternative to corticosteroid therapy. Pituitary. 2014;17(3):251–6. 10.1007/s11102-013-0498-9 23794123

[5] Cosman F, Post KD, Holub DA, Wardlaw SL. Lymphocytic hypophysitis. Report of 3 new cases and review of the literature. Medicine (Baltimore). 1989;68(4):240–56. 10.1097/00005792-198907000-00006 2661963

[6] Crock PA. Cytosolic autoantigens in lymphocytic hypophysitis. J Clin Endocrinol Metab. 1998;83(2):609–18. 10.1210/jcem.83.2.4563 9467582

[7] Deshpande V, Zen Y, Chan JK, et al. Consensus statement on the pathology of IgG4-related disease. Mod Pathol. 2012;25(9):1181–92. 10.1038/modpathol.2012.72 22596100

[8] Egloff B, Fischbacher W, von Goumoëns E. Lymphomatous hypophysitis associated with hypophyseal insufficiency. Schweiz Med Wochenschr. 1969;99(42):1499–502. 5363157

[9] Gleason TH, Stebbins PL, Shanahan MF. Lymphoid hypophysitis in a patient with hypoglycemic episodes. Arch Pathol Lab Med. 1978;102(1):46–8. 579597

[10] Goudie RB, Pinkerton PH. Anterior hypophysitis and Hashimoto’s disease in a young woman. J Pathol Bacteriol. 1962;83:584–5. 13900798

[11] Hadjigeorgiou GF, Lund EL, Poulsgaard L, Feldt-Rasmussen U, Rasmussen ÅK, Wegener M, Fugleholm K. Intrachiasmatic abscess caused by IgG4-related hypophysitis. Acta Neurochir (Wien). 2017;159(11):2229–33. 10.1007/s00701-017-3288-8 28799078

[12] Hasegawa Y, Matsumoto M, Kamimura A, Yamamoto M. A case of systematic lupus erythematosus with autoimmune hypophysitis. Nippon Naika Gakkai Zasshi. 1993;82(4):582–3. 8340666

[13] Hashimoto K, Asaba K, Tamura K, Takao T, Nakamura T. A case of lymphocytic infundibuloneurohypophysitis associated with systemic lupus erythematosus. Endocr J. 2002;49(6):605–10. 10.1507/endocrj.49.605 12625409

[14] Hashimoto M, Yanaki T, Nakahara N, Masuzawa T. Lymphocytic adenohypophysitis: an immunohistochemical study. Surg Neurol. 1991;36(2):137–44. 10.1016/0090-3019(91)90232-x 1891758

[15] Hume R, Roberts GH. Hypophysitis and hypopituitarism: report of a case. Br Med J. 1967;2(5551):548–50. 10.1136/bmj.2.5551.548 PMC1842464 6023990

[16] Khosroshahi A, Bloch DB, Deshpande V, Stone JH. Rituximab therapy leads to rapid decline of serum IgG4 levels and prompt clinical improvement in IgG4-related systemic disease. Arthritis Rheum. 2010;62(6):1755–62. 10.1002/art.27435 20191576

[17] Khosroshahi A, Carruthers MN, Deshpande V, Unizony S, Bloch DB, Stone JH. Rituximab for the treatment of IgG4-related disease: lessons from 10 consecutive patients. Medicine (Baltimore). 2012;91(1):57–66. 10.1097/MD.0b013e3182431ef6 22210556

[18] Kiaer W, Norgaard JO. Granulomatous hypophysitis and thyroiditis with lymphocytic adrenalitis. Acta Pathol Microbiol Scand. 1969;76(2):229–38. 5373630

[19] Koide H, Shiga A, Komai E, et al. Prednisolone-responsive postpartum IgG4-related hypophysitis. Intern Med. 2018;57(3):367–75. 10.2169/internalmedicine.8446-16 PMC5827318 29093382

[20] Lack EE. Lymphoid “hypophysitis” with end organ insufficiency. Arch Pathol. 1975;99(4):215–9. 1115687

[21] Leporati P, Landek-Salgado MA, Lupi I, Chiovato L, Caturegli P. IgG4-related hypophysitis: a new addition to the hypophysitis spectrum. J Clin Endocrinol Metab. 2011;96(7):1971–80. 10.1210/jc.2010-2970 PMC3135201 21593109

[22] Li JY, Lai PH, Lam HC, Lu LY, Cheng HH, Lee JK, Lo YK. Hypertrophic cranial pachymeningitis and lymphocytic hypophysitis in Sjögren’s syndrome. Neurology. 1999;52(2):420–3. 10.1212/wnl.52.2.420 9932974

[23] Mazzone T, Kelly W, Ensinck J. Lymphocytic hypophysitis. Associated with antiparietal cell antibodies and vitamin B12 deficiency. Arch Intern Med. 1983;143(9):1794–5. 10.1001/archinte.143.9.1794 6615105

[24] Nishiki M, Murakami Y, Koshimura K, Sohmiya M, Tanaka J, Yabe S, Kobayashi I, Kato Y. A case of autoimmune hypophysitis associated with asymptomatic primary biliary cirrhosis. Endocr J. 1998;45(5):697–700. 10.1507/endocrj.45.697 10395252

[25] Nussbaum CE, Okawara SH, Jacobs LS. Lymphocytic hypophysitis with involvement of the cavernous sinus and hypothalamus. Neurosurgery. 1991;28(3):440–4. 10.1097/00006123-199103000-00019 2011230

[26] Patel MC, Guneratne N, Haq N, West TE, Weetman AP, Clayton RN. Peripartum hypopituitarism and lymphocytic hypophysitis. QJM. 1995;88(8):571–80. 7648244

[27] Piñol V, Cubiella J, Navasa M, Fernández J, Halperin I, Bruguera M, Rodés J. Autoimmune hepatitis associated with thyroiditis and hypophysitis. A case report. Gastroenterol Hepatol. 2000;23(3):123–5. 10804689

[28] Sobrinho-Simões M, Brandão A, Paiva M, Vilela B, Fernandes E. Carneiro-Chaves F (1985) Lymphoid hypophysitis in a patient with lymphoid thyroiditis, lymphoid adrenalitis, and idiopathic retroperitoneal fibrosis. Arch Pathol Lab Med. 1985;109(3):230. 3838452

[29] Uccella S, Amaglio C, Brouland J-P, Bianconi E, Ippolito S, Messerer M, Rouiller N, Tanda ML, Sessa F, La Rosa S. Disease heterogeneity in IgG4-related hypophysitis: report of two histopathologically proven cases and review of the literature. Virchows Arch. 2019;475(3):373–81. 10.1007/s00428-019-02564-2 30911814

[30] van der Vliet HJJ, Perenboom RM. Multiple pseudotumors in IgG4-associated multifocal systemic fibrosis. Ann Intern Med. 2004;141(11):896–7. 10.7326/0003-4819-141-11-200412070-00033 15583245

[31] Ward L, Paquette J, Seidman E, Huot C, Alvarez F, Crock P, Delvin E, Kämpe O, Deal C. Severe autoimmune polyendocrinopathy-candidiasis-ectodermal dystrophy in an adolescent girl with a novel AIRE mutation: response to immunosuppressive therapy. J Clin Endocrinol Metab. 1999;84(3):844–52. 10.1210/jcem.84.3.5580 10084559

[32] Yuen KCJ, Moloney KJ, Mercado JU, Rostad S, McCullough BJ, Litvack ZN, Delashaw JB, Mayberg MR. A case series of atypical features of patients with biopsy-proven isolated IgG4-related hypophysitis and normal serum IgG4 levels. Pituitary. 2018;21(3):238–46. 10.1007/s11102-017-0852-4 29248982

